# Characterizations of botanical attractant of *Halyomorpha halys* and selection of relevant deorphanization candidates via computational approach

**DOI:** 10.1038/s41598-022-07840-x

**Published:** 2022-03-09

**Authors:** Yong-Zhi Zhong, Ming-Hui Xie, Cong Huang, Xue Zhang, Li Cao, Hao-Liang Chen, Feng Zhang, Fang-Hao Wan, Ri-Chou Han, Rui Tang

**Affiliations:** 1 Anhui-CABI Joint Laboratory for Agricultural Pest Control, Institute of Plant Protection and Agro-products Safety, Anhui Academy of Agricultural Sciences, Hefei, 230031 People’s Republic of China; 2 Guangdong Key Laboratory of Animal Conservation and Resource Utilization, Guangdong Public Laboratory of Wild Animal Conservation and Utilization, Institute of Zoology, Guangdong Academy of Sciences, Guangzhou, 510260 People’s Republic of China; 3 MARA-CABI Joint Laboratory for Bio-Safety, Institute of Plant Protection, Chinese Academy of Agricultural Sciences, 2 Yuanmingyuan West Road, Beijing, 100193 People’s Republic of China; 4 Shenzhen Branch, Guangdong Laboratory for Lingnan Modern Agriculture, Genome Analysis Laboratory of the Ministry of Agriculture, Agricultural Genomics Institute at Shenzhen, Chinese Academy of Agricultural Sciences, Shenzhen, 518120 People’s Republic of China; 5 College of Plant Health and Medicine, Qingdao Agricultural University, Qingdao, 266109 People’s Republic of China; 6 State Key Laboratory for Biology of Plant Diseases and Insect Pests, Institute of Plant Protection, Chinese Academy of Agricultural Sciences, Beijing, 100193 People’s Republic of China

**Keywords:** Chemical ecology, Computational biology and bioinformatics

## Abstract

*Halyomorpha halys* has been recognized as a global cross-border pest species. Along with well-established pheromone trapping approaches, there have been many attempts to utilize botanical odorant baits for field monitoring. Due to sensitivity, ecological friendliness, and cost-effectiveness for large-scale implementation, the selection of botanical volatiles as luring ingredients and/or synergists for *H. halys* is needed. In the current work, botanical volatiles were tested by olfactometer and electrophysiological tests. Results showed that linalool oxide was a potential candidate for application as a behavioral modifying chemical. It drove remarkable attractiveness toward *H. halys* adults in Y-tube assays, as well as eliciting robust electroantennographic responsiveness towards antennae. A computational pipeline was carried out to screen olfactory proteins related to the reception of linalool oxide. Simulated docking activities of four *H. halys* odorant receptors and two odorant binding proteins to linalool oxide and nerolidol were performed. Results showed that all tested olfactory genes were likely to be involved in plant volatile-sensing pathways, and they tuned broadly to tested components. The current work provides insights into the later development of field demonstration strategies using linalool oxide and its molecular targets.

## Introduction

We have been fighting against agricultural and forestry pest insects for centuries. Among them, invasive, cross-border species are drawing emerging attention along with the changing of global climate, agronomic changes, and human activities^1^. Due to lack of local natural enemies, invasive pest can easily spread and cause severe damage on crop/vegetation over the world^2^. Despite intensive management efforts, choices for monitoring and control are limited. Years of experience have resulted in several promising strategies such as biological control, transgenic variation, and more importantly, ecological approaches *e.g.*, luring technology based on understanding of key volatile cues and chemical communications of target species^3–5^. Efforts to develop effective attractants have been huge, but the development of trap technology can result in more environmentally friendly management strategies and are worth the cost^6,7^.

The brown marmorated stink bug, *Halyomorpha halys* (Stål) (Hemiptera: Pentatomidae), is native to Eastern Asia and has spread globally with localized strains identified in North America and Europe. Recent occurrences of this species have been reported also in Oceania and South America^8^. Over 120 host plants from fruit crops, vegetables, ornamentals, shrubs, and forest trees were confirmed to be damaged by this polyphagous pest huge annual crop losses have been reported^9^. In order to conduct monitoring and control methods, aspects of chemical ecology have been well tackled for *H. halys* and its sibling species^10–12^. Utilizations of aggregation pheromones, alarm pheromones, attract-and-kill, push–pull strategies along with integrated management approaches are under development^13–17^. Meanwhile, a monitoring protocol for *H. halys* has matured since the two-component attractant was developed from Pentatomidae pheromone components^7,9,18,19^. Nevertheless, new studies are emerging to identify and improve synergism of additional chemicals with undergoing attractants, as well as to increase the cost-effectiveness of the baiting methods^20^.

Of the semiochemical research done on *H. halys* during 2019–2021 (Table S1), most studies (64.7%) explored using pheromone and synergist components of *H. halys* or other hemipteran species. The major focuses of such works were evaluating traps, densities, periods, pheromone recipes, trapping-based IPMs, etc.^20–31^. The second most tackled approach was physical interventions, including photoselective exclusion netting, and vibrational signals, which compiled 41.2% of recent works^32,33^. Host plants were evaluated in some works for direct trapping of *H. halys* (17.6%)^22,26,34–36^. Relatively few studies concerned botanical volatile attractant development (11.8%). However, some novel attractant and/or synergistic cues were identified in these works, including hexanal, (±)-*α*-pinene, (−)-sabinene, and others^34,37^. Host plant volatiles may expand the trapping ability of current lures, and they are usually good candidates for pest baiting by screening the key ingredients and the best combinations. To date, most host plant odorants are designed as synergists for already existing lure products, in order to increase luring efficiency and/or lower the costs for large scale implementations^38–40^. Attempts for improvements and optimizations of undergoing attractants are still needed for pest management^41,42^.

It is challenging to start from scratch to select natural products as attractants or agonists for pests^43^. One cost-effective strategy was to focus on available host cues which have been proved bioactive toward common insect species^44^. The chemical linalool oxide, which serves as a generalist plant-based lure, has been applied toward many insects^45^. As a tetrahydrofuran, linalool oxide was presented in various orchard fruits as a universal flavor within volatile blends^46^. This component was implemented as an odorant bait ingredient for different insect families including malaria vectors, lepidopteran adults, and beetles^46–48^. It could be a potential additive for odorant baiting in stink bugs, but has hardly been tested toward the family Pentatomidae. We have evaluated linalool oxide in a field trial, and this component exhibited equivalent attractiveness to *H. halys* compared to an odor bait mixture^49^. However, the reception of this volatile by *H. halys* olfaction was still elusive.

Chemical communications among *H. halys*, plants, and natural enemies have been studied, and a lot of background information has been reported in terms of the ecological and molecular basis of olfaction^50–52^. However, works need to be done on localization of the peripheral and central neural pathways in which key volatiles and proteins interact with each other. We have previously used a DREAM-like method (Deorphanization of receptors based on expression alterations in mRNA levels) to pre-screen odorant binding proteins (OBPs) for sensing *E*-2-decenal in *H. halys*^53,54^. Another strategy to evaluate candidate volatiles is to conduct homological alignments, so that tested spectra for both olfactory genes and ligand volatiles can be narrowed^55^. Benefiting from simulation technology, molecular docking has been widely used to assess bindings of olfactory proteins to botanical ligands in insects^56,57^. In summary, based on the continuously improving modeling database for insect receptor proteins, in silico methods have provided us with trial and error opportunities before lab tests^58,59^.

In the current work, a series of indoor olfactometer assays and electrophysiological tests were done to further select additional ingredients for improvements in bait recipes for *H. halys*. Homological selection and simulated molecular docking were carried out to locate potential *H. halys* olfactory receptors (HahlORs) and HhalOBPs for screened chemical components, in order to provide insights to deorphanization attempts in the future.

## Materials and methods

### Insects

Nymphs and adults of *H. halys* were obtained from laboratory colonies at the Institute of Plant Protection and Agro-products Safety, Anhui Academy of Agricultural Sciences, Hefei. They were continuously reared on a diet of organic green beans (*Phaseolus vulgaris* L.) and corn (*Zea mays* L.) in rearing cages (60 × 60 × 60 cm) at 25 ± 1 °C, 65 ± 5% RH and 16 L: 8 D photoperiod. Insects were fasted for 1–2 h prior to the tests.

### Chemicals

Synthetic standard chemicals used within the study for bioassays and electrophysiological tests were commodities including n-hexane (95%, Sigma-Aldrich, St. Louis, MO, USA) as solvent, and linalool oxide (99%, Sigma-Aldrich, St. Louis, MO, USA), nerolidol (99%, Sigma-Aldrich, St. Louis, MO, USA), methyl (*E*,*E*,*Z*)-2,4,6-decatrienoate (95%, Sigma-Aldrich, St. Louis, MO, USA), and n-dodecane (99%, Sigma-Aldrich, St. Louis, MO, USA) as treatments.

### Olfactometer assay

Olfactometer assays were done using a Y-tube system which was previously described^17^. Parameters for the Y-tube were: stem length at 30 cm, arm length at 20 cm, stem diameter at 3 cm, arm diameter at 2.5 cm, and arm angle at 90°. Airflow was constantly fixed at 0.5 L/min with purification and humidify done by successive connections to activated carbon and double distilled water. Laddered solutions were done by mixing standard chemicals with n-hexane solvent. The control arm was set by providing the same volume of n-hexane solvent to compare with each treatment. Single *H. halys* adult was introduced from the end of the stem tube and allowed to choose within 5 min during each trial. All tests were done during scotophase under infrared light at 25 ± 2 °C and 40–60 RH. A total of 30 replicates were done for each chemical at each dosage toward each gender.

### Electroantennogram (EAG) recording

EAG was used to identify electrophysiological activities of adult *H. halys* to linalool oxide. Each *H. halys* antenna was prepared following standard procedures by cutting the tip and base of the antenna and immediately mounting the excised antenna between two ends of a recording probe (Ockenfels SYNTECH GmbH, Buchenbach, Germany). The other end of the recording probe was directly connected via an interface box to a signal acquisition interface board (IDAC 2; Ockenfels SYNTECH GmbH, Buchenbach, Germany). Stimulations were manually driven by a gas stimulator (CS-55, Ockenfels SYNTECH GmbH, Buchenbach, Germany). Linalool oxide was tested at dosages of 1 μg, 10 μg, and 100 μg, respectively, and n-hexane was used as control. Each stimulation record contained successive measurements of air–control–treatment–control–air. Continuous air flow was set at 150 mL/min, and stimulate flow velocity was 20 mL/min for 0.1 s.

n-Hexane (95%, Sigma-Aldrich, St. Louis, MO, USA) was used as control. One μg/μl (*E*)-2-decenal (95%, Sigma-Aldrich, St. Louis, MO, USA) and the measure dosage was 10 μg, and the measure order of antenna was air–n-hexane–(*E*)-2-decenal. The replication was 10, direct voltage was 2 mv, continuous flow velocity was 150 ml/min, stimulate flow velocity was 20 ml/min, stimulation time was 0.1 s, and stimulus intervals was 10 s. Raw data of voltages were transferred by: Relative response value (mV) = sample response value (mV)—control response value (mV) before statistical analysis was done.

### Olfactory gene characterization

Gene families of OBPs and ORs of *H. halys* were collected from previous reported works^53,60–62^. Translated amino acid sequences (Dataset S1) of selected ORs from *Halyomorpha halys*, *Apolygus lucorum*, *Sogatella furcifera*, *Cimex lectularius*, *Drosophila melanogaster*, *Bombyx mori*, *Mythimna separata*, and *Helicoverpa armigera* were firstly aligned with MUSCLE and phylogenetic tree was developed using the Neighbor-Joining method^63^ in MEGA-X 10.1.8 software^64^ before formatted with FigTree v 1.4.4^65^. Structural predictions were done using SWISS-MODEL (https://swissmodel.expasy.org, Basel, Switzerland). Amino acid alignment was done using PRALINE multiple sequence alignment (https://www.ibi.vu.nl/programs/pralinewww, Amsterdam, The Netherlands).

### Simulated molecular docking

Docking studies were done to predict binding of selected *H. halys* olfactory proteins toward linalool oxide and nerolidol, respectively. All known 30 OBPs and 4 ORs of *H. halys* were SWISS-MODEL-ed and assessed by GMQE and QMEAN values. Specifically, the confidential interval for GMQE was set at 0–1, and QMEAN was set at [− 4, 0]. Higher GMQE values indicated more promising modeling, and lower QMEAN values indicated better binding possibilities of ligands and the selected proteins. For OBPs, a lower cutting threshold of 30% identity was used to initially screen from 30 proteins before docking was done.

Three-dimensional structures of tested volatiles were downloaded from Pubchem (https://pubchem.ncbi.nlm.nih.gov). Data were transferred to PDB formats via OpenBabel V3.0^66^ before energies of ligand structures were minimized by Molecular Operating Environment (MOE; CCG ULC., Montreal, Canada). The docking algorithm was conducted by using AutoDockTools (ADT)^67^. Docking affinities of selected proteins to ligands were automatically evaluated by ADT. Docking results were visualized and exported as vector images by PyMol (The PyMOL Molecular Graphics System, Version 2.0 Schrödinger, LLC.) before edited in Adobe Illustrator CS6 software (Adobe, San Jose, CA, USA).

### Statistics and data processing

Comparison of means was done using SPSS with GLM and Tukey HSD at α = 0.05. Counts data was compared with *Chi*-square test at α = 0.05. All statistics were carried out using IBM SPSS Statistics 22.0.0 (SPSS, Chicago, IL, USA). Bar and plot charts were developed using Prism 5 for Windows ver. 5.01 (GraphPad software, San Diego, CA, USA). Correlation matrix was developed with Statgraphics Centurion XVII (Statpoint Technologies, Inc., VA, USA).

### Data accessibility

All described data in this work have been included in the manuscript and online supplementary materials (Table S1–S3, Dataset S1).

## Results

### Behavioral valence of *H. halys* to selected allelochemicals

**Figure 1:**
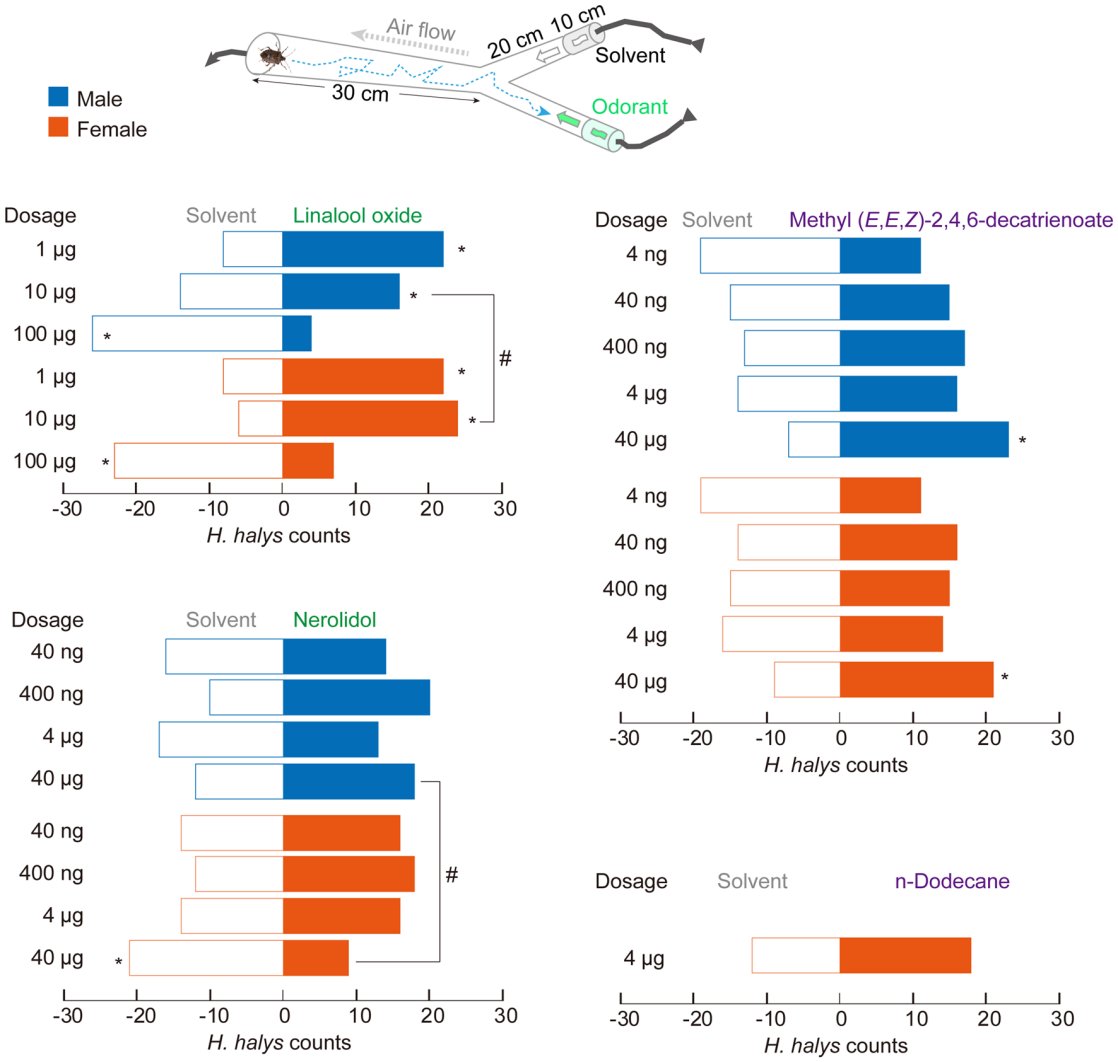
Behavioral valence of *H. halys* to various tested volatiles. Results of Y-tube assays with *H. halys* by applied selected botanical and pheromone-related chemicals. Asterisk indicates significant choice preferences of *H. halys* to either tested chemicals or solvent control (*Chi*-square test, *P* < 0.05, n = 30 for each gender at each dosage toward each treatment). Hashtag indicates significant choice preferences between genders to the same dosage treatment (*Chi*-square test, *P* = 0.0285 for linalool oxide at 10 μg and *P* = 0.0195 for nerolidol at 40 μg).

Within all four tested chemicals, linalool oxide elicited significant choice behaviors at the lowest dosages (Fig. 1). Both female and male adults of *H. halys* chose linalool oxide in the Y-tube assays when the chemical was applied at 1 µg and 10 µg. While higher dosages of 100 µg drove contrary choice results in both genders, reflecting the potential repellency role of linalool oxide at high dosages. Other tested chemicals did not drive observable choice behaviors until they were applied at 40 µg dosages. Furthermore, linalool oxide at 10 μg and nerolidol at 40 μg stimulated significant gender-biased behavioral preferences, showing that female *H. halys* adults were more sensitive than males under these tested dosages (Fig. 1). In sum, *H. halys* adults are most sensitive toward linalool oxide within all tested chemicals.

### Electrophysiological responses of *H. halys* antennae toward linalool oxide

**Figure 2:**
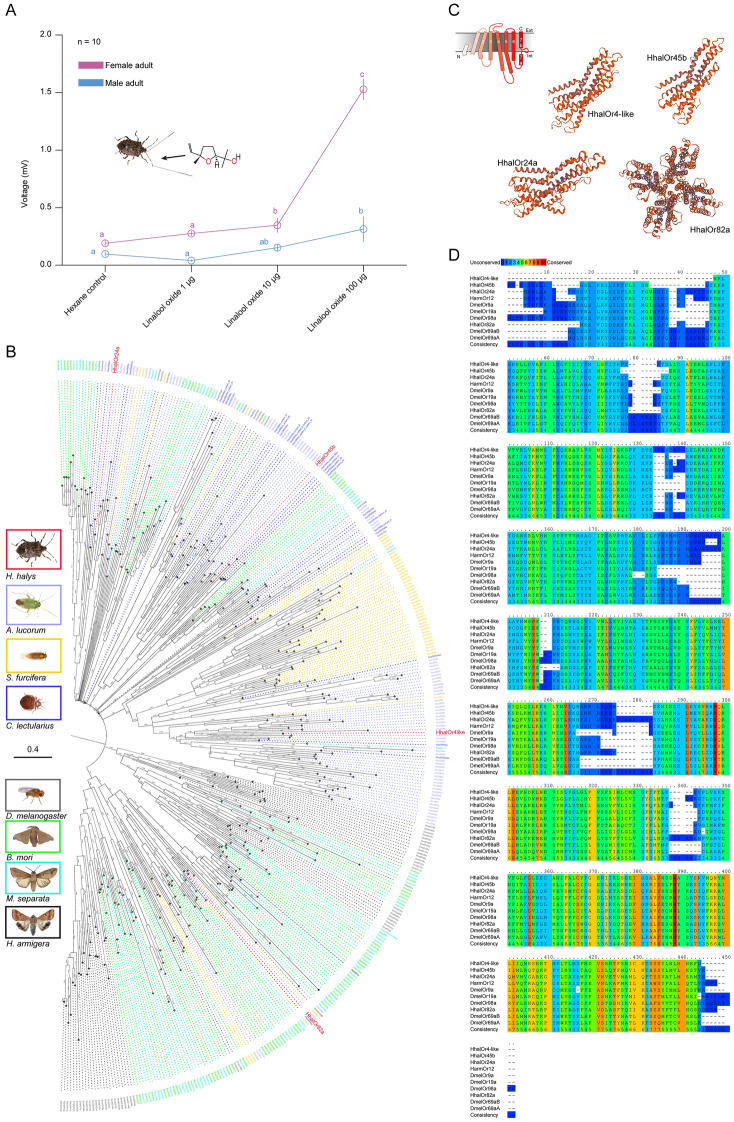
Olfactory evidences of *H. halys* in sensing linalool oxide. (**A**) Results of electroantennogram tests with linalool oxide. Lower-case letters indicate significant differences among tested dosages in either male or female adults. (GLM and Tukey HSD multiple comparison. *P* < 0.05. Error bars indicate ± s.e.m.) (**B**) Phylogenetic analysis of putative linalool sensing odorant receptors of *H. halys* by referring to ORs from *A. lucorum*, *S. furcifera*, *C. lectularius*, *D. melanogaster*, *B. mori*, *M. separata*, and *H. armigera*. The evolutionary history was inferred by using the Maximum Likelihood method and the JTT matrix-based model. The tree with the highest log likelihood (− 2,980,075.06) is shown. Initial tree(s) for the heuristic search were obtained automatically by applying Neighbor-Join and BioNJ algorithms to a matrix of pairwise distances estimated using the JTT model, and then selecting the topology with superior log likelihood value. This analysis involved 374 amino acid sequences. There were a total of 1344 positions in the final dataset. Evolutionary analyses were conducted in MEGA X. (**C**) Structural predictions of four putative linalool sensing ORs in *H. halys*, with modeling re-constructed referring to known Cryo-EM structures of insect ORco (*Apocrypta bakeri* ORco: 6c70.1.A) Schematic shows representative 7-TMD structure of insect OR. All four ORs showed 7-TMD structures and potentially tetramer binding activities. Predictions were done with SWISS-MODEL. (**D**) Alignment of the four *H. halys* ORs with referring to most related linalool sensing ORs in *Drosophila* and cotton bollworm. Conservations of amino acid residues were indicated with colors.

The resolutions of EAG tests were lower for linalool oxide compared with the behavioral assays (Fig. 2A). Female antennae showed responsiveness to linalool oxide at 10 µg dosages and the responding level increased dramatically along with the increase in dosage. On the other hand, male antennae only started to respond to linalool oxide when applied at 100 µg (Fig. 2A). Overall, gender bias was observed in the EAG assays, as also shown in the Y-tube assays that females were more sensitive than male *H. halys* adults (Figs. 1, 2A).

### Characterization of putative odorant receptors for linalool oxide

A total of four putative receptors for linalool oxide were screened by BLASTP against known linalool oxide sensing ORs in Drosophila and *H. armigera*. Reference ORs were HarmOr12, DmelOr19a, DmelOr69aB, DmelOr69aA, DmelOr98a, and DmelOr9a, which, were reported to have tuned to ( ±)-linalool and/or linalool oxide stimuli^68,69^. The selected HhalOR24a, HhalOr45b, HhalOr82a, and HhalOr4-like were separated into four major clusters. Among them, HhalOr82a was more similar with lepidopteran ORs other than hemipteran ones (Fig. 2B). Structural predictions of these four proteins all showed a representative 7-TMD structure of insect ORs (Fig. 2C), and they were able to form the tetramer structure which was proved to be the functional basis of ORs. When the four ORs were aligned with reference ORs, it showed that they were not conserved in the N– part. However, much more conservation was observed during the C– end (Fig. 2D). Since all referred insect ORs to linalool oxide were broadly tuning ORs toward plant odorants, we carried out docking simulations using the four HhalORs in order to investigate *H. halys* attractiveness to this botanical volatile at the molecular level.

### Simulated docking to linalool oxide and nerolidol

**Figure 3:**
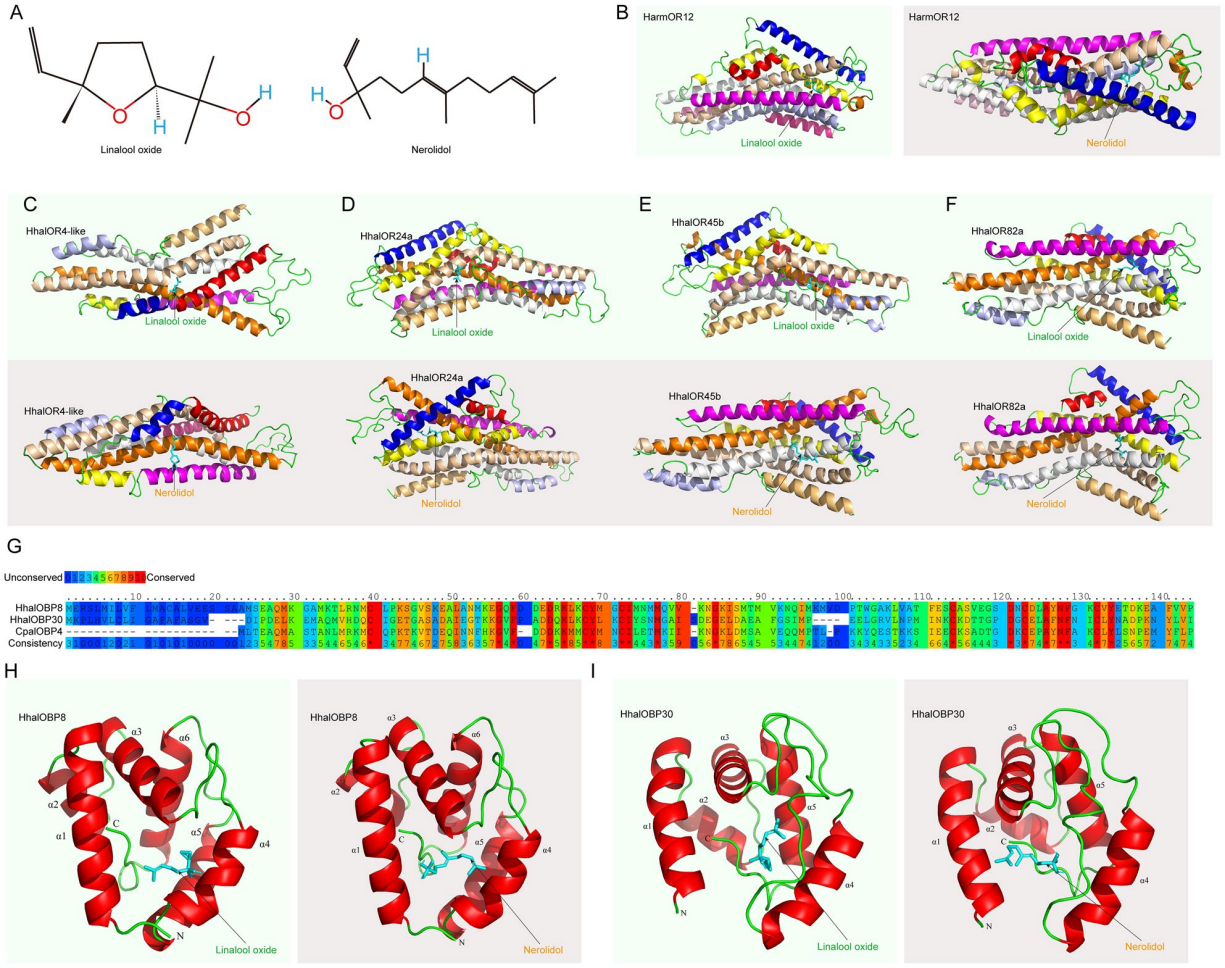
Molecular docking studies of *H. halys* olfactory proteins to linalool oxide and nerolidol. (**A**) Chemical structures of linalool oxide (left) and nerolidol (right). (**B**) Schematics showing docking poses of HarmOR12 binding with linalool oxide (left) and nerolidol (right), respectively. (**C–F**) Schematics showing docking poses of HhalOR4-like (C), HhalOR24a (D), HhalOR45b (E), and HhalOR82a (F) binding with linalool oxide (up) and nerolidol (down), respectively. (**G**) Sequence alignment between HhalOBP8, HhalOBP30, and CpalOBP4 (*Chrysopa pallens* OBP4: 6jpm.1.A). Conservations of amino acid residues were indicated with colors. Identities of HhalOBP8 with CpalOBP4 was 36.97%, and for HhalOBP30 and CpalOBP4 it was 33.04%. (**H–I**) Schematics showing docking poses of HhalOBP8 (H) and HhalOBP30 (I) binding with linalool oxide (left) and nerolidol (right), respectively.

Simulated docking studies were conducted using HhalOR4-like, HhalOR24a, HhalOR45b, and HhalOR82a, comparing with the parameters of HarmOR12 (Fig. 3A–F, Table S2). The tested ligands were the tetrahydrofuran linalool oxide and the sesquiterpene alcohol nerolidol (Fig. 3A). The *H. armigera* OR12 was checked for binding affinity to linalool oxide by stimulated docking as reference. It showed that binding energies for HarmOr12 with nerolidol was − 4.25, and it was − 3.26 with linalool-oxide (Fig. 3B, Table S 2). Similar results were observed for HhalOR24a, HhalOR45b, and HhalOR82a, which presented lower binding energies for nerolidol than for linalool-oxide (Fig. 3D–F, Table S 2). While HhalOR4-like exhibited better predicted binding affinity with linalool-oxide (− 3.22) than nerolidol (− 2.97) (Fig. 3C). Among, HhalOR82a was predicted to be the best matched receptor for nerolidol with binding energy at − 4.49. For linalool-oxide, the best predicted receptors were HhalOR4-like and HhalOR82a, both with a binding energy at − 3.22 (Table S 2).

In order to draw a promising conclusion, investigations on OBPs were also done with similar protocols and algorithms. However, among 30 HhalOBPs, we only identified two qualified OBPs namely HhalOBP8 and HhalOBP30 which had > 30% identities with reference model (*Chrysopa pallens* OBP4: 6jpm.1.A; CpalOBP4). Both HhalOBP8 and HhalOBP30 were typical 6-C OBPs as CpalOBP4, and the identities for comparing with CpalOBP4 were 36.97% for HhalOBP8 and 33.04% for HhalOBP30, respectively (Fig. 3G). We have observed that binding energies for HhalOBP8 with nerolidol was − 4.81, and it was − 3.71 for HhalOBP30 with nerolidol. For another ligand linalool oxide, simulated binding energies were − 3.17 with HhalOBP8 and HhalOBP30, respectively (Fig. 3H,I, Table S3). Due to the results, it was suggested that both HhalOBP8 and HhalOBP30 could be general OBPs which were involved in plant odorant sensation. HhalOBP8 had better binding potential to nerolidol than HhalOBP30 did.

## Discussion

Shelter plants of insect pests can exhibit various volatile compounds^68^. In the current study, we revisited a series of botanical ingredients, and revealed attractiveness of linalool oxide under mid dosages during bioassays and electrophysiological tests toward *H. halys* adults, especially females. Higher dosages of linalool oxide and nerolidol repelled the adults from the odorant sources. As a common additional ingredient in bait formulations for moths, linalool oxide was assessed by simulated molecular docking with phylogenetically selected *H. halys* ORs and OBPs. The computational results showed that similar binding affinities were found for HarmOR12 reference and four *H. halys* ORs including HhalOR4-like, HhalOR24a, HhalOR45b, and HhalOR82a. Furthermore, HhalOBP8 and HhalOBP30 out of 30 HhalOBPs showed moderate binding potentials to linalool oxide and nerolidol. These six olfactory genes should be prioritized for further functional tests in order to identify relevant reception basis for sensing linalool oxide and nerolidol in *H. halys*.

### Utilization of hemipteran olfaction

Pheromone lures have been one of the most popular monitoring methods for *H. halys* and other Pentatomidae around the globe^70^. Many aggregation pheromones of this family share the bisabolene backbone including the *H. halys* which employ a two-component recipe of 3S6S7R10S-murgantiol and 3R6S7R10S-murgantiol^7^. This recipe was improved later by adding synergists such as methyl (2*E*,4*E*,6*Z*)-2,4,6-decatrienoate (MDT)^20,71^. The downside of such practices was that they represented species-specific biomarker volatiles that enhance aggregation and retention of *H. halys*, and this may result in heavier crop damage at the implementation site^72^. As this species of stink bug had high sensitivity toward certain botanical volatile resources, cost-effective alternatives/additives of the *H. halys* odor bait may be developed from host plant emissions^34,37^. Nevertheless, most of the trapping works for *H. halys* have not considered plant volatiles for olfactory perception. Compared to other Hemiptera to which molecular regulations based on olfaction have been applied, works for *H. halys* are relatively rare^62,73,74^. Future studies may involve identifications of key host cues, their molecular targets, and reliable implementation methods, *e.g.*, the RNA interference approach^75^. Furthermore, the mechanism by which key volatile signals are coded through central olfactory systems of this stink bug is still unknown and a fascinating area to explore.

### Linalool oxide as additional ingredient for odorant bait

The tetrahydrofuran linalool oxide was reported to have enriched botanical bait formulations, and originated from plant volatiles/essence^76,77^. Lures containing linalool oxide have been widely applied for trapping insects including moths, mosquitoes, and beetles^47,48,78^. As a common emission from natural botanical products, linalool oxide was found to be related to plant injury^77,78^. The secondary metabolite role of linalool oxide has implied its potential functions in plant defense^79^. In fact, this component has also been used as a control reagent for houseflies and coffee bugs^80,81^. For *H. halys* and other stink bugs, attraction by linalool oxide has not yet been reported. Literature has shown that this species is behaviorally modified by plant essential oils, which have presented this chemical in mixed volatile blends^12^. Some works have reported that selected key ingredients of volatiles may work better than a full spectrum of plant volatile blend^82^. It may support that linalool oxide has the potential to be a vital addition for optimization of current commercial luring recipes for *H. halys* as this chemical has outperformed other tested components in the Y-tube assays. The results from the current study have raised the possibility that linalool oxide can be used as an ecological insect behavioral modifying chemical in stink bugs, as is revealed in lepidopteran and dipteran species. Future field trials and implementation of fully-established botanical blend recipes for testing the final effectiveness of artificial baiting approaches for *H. halys* could benefit from screening on more terpenoids, tetrahydrofurans, esters, and aromatics.

### Narrowed-down spectra for receptor protein deorphanization

One important move in the field of chemical ecology is to identify functional genes (mostly receptors) for understanding the olfactory reception of selected bioactive odorants, or “deorphanization”^55^. The so-called reversed chemical ecology sought to solve this matter by providing peripheral coding information for later sorting signaling from brain innervated patterns in higher neuropils of insects^83^. However, most of the volatile ligands did not activate all receptors in a species. A sensing spectrum provided by an OR limits the firing pattern of the corresponding odorant sensory neuron and thus influenced behavioral outputs of insects^84^. All in all, if a previous selection could be done, it would not always be necessary to functionally demonstrate all existing receptor genes against a known volatile ligand. Because thousands of candidate plant volatiles would generate endless research, and we would like our hypothesis tested swiftly and accurately by saving time, labor, and cost. Fortunately, there are several approaches for narrowing down candidate spectra of ORs and OBPs ready for deorphanization. For example, DREAM technology was developed in *Mammalia* and *Drosophila* and was reported to have worked in stink bugs^53,85,86^, and we successfully utilized this method to allocate pheromone OBPs in *H. halys*^54^. Benefiting from abundant gene annotations, another straight-forward method was to conduct homology alignment combined with molecular docking simulation. Works have shown that docking results could fit well with functional tests. Thus, a total of two HhalOBPs and 4 HhalORs were highlighted using this approach from the current research, and can become vital candidates for testing binding and reception of linalool oxide as well as nerolidol in further studies.

### Combinatorial coding by multiple-receptor decision toward allelochemicals

It was intriguing that linalool oxide attracted *H. halys* with lower to middle range dosages while repelling them when applied at a high dosage. This phenomenon was identified in *Drosophila*, which employed a sensory switch to drive contrary behaviors in sour and salt reception^87,88^. It is possible that linalool oxide was coded via a combinatorial pathway through the olfactory systems in *H. halys*, as it has been shown in the vinegar fly. As a common botanical volatile component, linalool oxide was not likely to be involved in the labeled line circuits, which were mostly used by insects to decide life-and-death issues^89^. While higher concentrations in the air of linalool oxide could mean damage already done to the plants^77^, and this repellent modality may help this species to balance their populations within the distributed areas^78,90^. This potential ecological significance of dose may be useful for development of linalool oxide- and nerolidol-based push–pull strategies in a more precise way. On the other side, multiple pathways decision system by the insects’ olfaction involved several ORs and glomeruli in the neuropil^91,92^. In this work, selected OBPs and ORs shared similar docking affinities with linalool oxide and nerolidol ligands, which also implied that the olfactory reception pathways for these components involved more than one receptor to be functionally operated^69^. Furthermore, tuning spectra for linalool oxide of the *H. halys* ORs may also be broad, as it has been shown in docking simulation results, indicating that additional botanical behavioral modification chemicals may exist.

## Supplementary Information

Dataset S1.

Supplementary Tables.
