# Supplementary material for: Characterizations of botanical attractant of Halyomorpha halys and selection of relevant deorphanization candidates via computational approach
Source: Sci Rep. 2022 Mar 9;12:4170. doi: 10.1038/s41598-022-07840-x (PMC8907264; doi:10.1038/s41598-022-07840-x)
Supplement: Supplementary file 2 — Supplementary Tables. [file 41598_2022_7840_MOESM2_ESM.docx]

*Supplementary materials for:*

**Characterizations of botanical attractant of *Halyomorpha halys* and selection of relevant deorphanization candidates via computational approach**

Yong-Zhi Zhong^#^, Ming-Hui Xie^#^, Cong Huang^#^, Xue Zhang, Li Cao, Hao-Liang Chen, Fang-Hao Wan, Ri-Chou Han, Rui Tang^*^

Contents:

**Table S1:** Reviewed recent literatures on luring of *H. halys*.

**Table S2:** Docking parameters of HhalORs and ligands.

**Table S3:** Docking parameters of HhalOBPs and ligands.

**Dataset S1**: FASTA format of amino acid sequences used in the phylogenic analysis (online submission)

**Table S1.** List of reviewed literatures on trapping applications for *H. halys*. Citations were listed in the end of this file. Numbers indicate yes = 1 and no = 0.

| Plant | Pheromone | Botanical | Physical | Reference |
| --- | --- | --- | --- | --- |
| 1 | 0 | 0 | 0 | Soergel et al., 2015 [1] |
| 0 | 0 | 1 | 0 | Leskey et al., 2015a [2] |
| 0 | 1 | 0 | 0 | Leskey et al., 2015b [3] |
| 0 | 1 | 1 | 0 | Morrison III et al., 2018 [4] |
| 0 | 0 | 0 | 1 | Leskey et al., 2015c [5] |
| 0 | 0 | 0 | 1 | Mazzoni et al., 2017 [6] |
| 0 | 1 | 0 | 0 | Kirkpatrick et al., 2019 [7] |
| 0 | 1 | 0 | 0 | Morrison III et al., 2015 [8] |
| 0 | 1 | 0 | 0 | Morrison III et al., 2017a [9] |
| 0 | 1 | 0 | 0 | Akotsen-Mensah et al., 2018 [10] |
| 1 | 0 | 0 | 0 | Nielsen et al., 2016 [11] |
| 0 | 1 | 0 | 1 | Rice et al., 2018a [12] |
| 0 | 1 | 0 | 0 | Morrison III et al., 2017b [13] |
| 0 | 1 | 0 | 0 | Rice et al., 2018b [14] |
| 0 | 1 | 0 | 0 | Short et al., 2017 [15] |
| 1 | 0 | 0 | 0 | Mathews et al., 2017 [16] |
| 0 | 1 | 0 | 0 | Blaauw et al., 2017 [17] |
| 0 | 1 | 0 | 0 | Acebes-Doria et al., 2018 [18] |
| 0 | 1 | 0 | 0 | Acebes-Doria et al., 2020 [19] |
| 0 | 1 | 0 | 0 | Tillman et al., 2017 [20] |
| 0 | 1 | 0 | 1 | Rice et al., 2017 [21] |
| 0 | 1 | 1 | 0 | Chase et al., 2018 [22] |
| 0 | 1 | 0 | 1 | Bae et al., 2017 [23] |
| 0 | 1 | 1 | 0 | Morrison III et al., 2019 [24] |
| 0 | 0 | 0 | 1 | Acebes-Doria et al., 2016 [25] |
| 0 | 1 | 0 | 1 | Suckling et al., 2019 [26] |

**Table S2.** Docking parameters of HhalORs and HarmOR12 toward tested ligands. Amino acid sequences were provided in Dataset S1.

| Gene | Ligands | binding energy | ligand efficiency | inhib constant | intermol energy | vdw hb desolv energy | electrostatic energy | total internal | torsional energy | unbound energy | clRMS | refRMS |
| --- | --- | --- | --- | --- | --- | --- | --- | --- | --- | --- | --- | --- |
| HhalOR4-like | Linalool-o | -3.22 | -0.27 | 4.4mM | -4.71 | -4.73 | 0.02 | -0.98 | 1.49 | -0.98 | 0 | 345.42 |
|  | Nerolidol | -2.97 | -0.19 | 6.64mM | -5.36 | -5.37 | 0.01 | -0.68 | 2.39 | -0.68 | 0 | 348.56 |
| HhalOR45b | Linalool-o | -3.06 | -0.26 | 5.68mM | -4.56 | -4.24 | -0.31 | -0.31 | 1.49 | -0.31 | 0 | 343.5 |
|  | Nerolidol | -3.49 | -0.22 | 2.75mM | -5.88 | -5.85 | -0.03 | -0.57 | 2.39 | -0.57 | 0 | 324.54 |
| HhalOR24a | Linalool-o | -2.74 | -0.23 | 9.82mM | -4.23 | -4.2 | -0.03 | -0.41 | 1.49 | -0.41 | 0 | 335.16 |
|  | Nerolidol | -4.61 | -0.29 | 415.84uM | -7 | -7.04 | 0.04 | 0.56 | 2.39 | 0.56 | 0 | 333.63 |
| HhalOR82a | Linalool-o | -3.22 | -0.27 | 4.36mM | -4.71 | -4.71 | 0 | -0.53 | 1.49 | -0.53 | 0 | 332.4 |
|  | Nerolidol | -4.49 | -0.28 | 507.37uM | -6.88 | -6.77 | -0.11 | 0.45 | 2.39 | 0.45 | 0 | 333.04 |
| HharmOR12 | Linalool-o | -3.26 | -0.27 | 4.05mM | -4.76 | -4.71 | -0.05 | -0.8 | 1.49 | -0.8 | 0 | 334.2 |
|  | Nerolidol | -4.25 | -0.27 | 767.95uM | -6.64 | -6.67 | 0.03 | 1.25 | 2.39 | 1.25 | 0 | 333.41 |

Linalool-o: Linalool oxide

**Table S3.** Docking parameters of HhalOBPs toward tested ligands. Amino acid sequences were provided in Dataset S1.

| Gene | Ligands | binding energy | ligand efficiency | inhib constant | intermol energy | vdw hb desolv energy | electrostatic energy | total internal | torsional energy | unbound energy | clRMS | refRMS |
| --- | --- | --- | --- | --- | --- | --- | --- | --- | --- | --- | --- | --- |
| HhalOBP8 | Linalool-o | -3.6 | -0.3 | 2.29mM | -5.09 | -5.08 | -0.02 | 0.11 | 1.49 | 0.11 | 0 | 29.85 |
|  | Nerolidol | -4.81 | -0.3 | 299.45uM | -7.19 | -7.18 | -0.02 | -0.58 | 2.39 | -0.58 | 0 | 29.84 |
| HhalOBP30 | Linalool-o | -3.17 | -0.26 | 4.76mM | -4.66 | -4.6 | -0.06 | -0.31 | 1.49 | -0.31 | 0 | 28.45 |
|  | Nerolidol | -3.71 | -0.23 | 1.9mM | -6.1 | -6.1 | 0 | -0.22 | 2.39 | -0.22 | 0 | 29.41 |

Linalool-o: Linalool oxide

**References:**

**[1]** Soergel D C, Ostiguy N, Fleischer S J, et al. Sunflower as a potential trap crop of *Halyomorpha halys* (Hemiptera: Pentatomidae) in pepper fields[J]. Environmental entomology, 2015, 44(6): 1581-1589. https://doi.org/10.1093/ee/nvv136

**[2]** Leskey T C, Khrimian A, Weber D C, et al. Behavioral responses of the invasive *Halyomorpha halys* (Stål) to traps baited with stereoisomeric mixtures of 10, 11-epoxy-1-bisabolen-3-ol[J]. Journal of Chemical Ecology, 2015, 41(4): 418-429. https://doi.org/10.1007/s10886-015-0566-x

**[3]** Leskey T C, Agnello A, Bergh J C, et al. Attraction of the invasive *Halyomorpha halys* (Hemiptera: Pentatomidae) to traps baited with semiochemical stimuli across the United States[J]. Environmental entomology, 2015, 44(3): 746-756. https://doi.org/10.1093/ee/nvv049

**[4]** Morrison III W R, Allen M K, Leskey T C. Behavioural response of the invasive *Halyomorpha halys* (Hemiptera: Pentatomidae) to host plant stimuli augmented with semiochemicals in the field[J]. Agricultural and forest entomology, 2017, 20(1): 62-72. https://doi.org/10.1111/afe.12229

**[5]** Leskey T C, Lee D H, Glenn D M, et al. Behavioral responses of the invasive *Halyomorpha halys* (Stål)(Hemiptera: Pentatomidae) to light-based stimuli in the laboratory and field[J]. Journal of Insect Behavior, 2015, 28(6): 674-692. https://doi.org/10.1007/s10905-015-9535-z

**[6]** Mazzoni V, Polajnar J, Baldini M, et al. Use of substrate-borne vibrational signals to attract the brown marmorated stink bug, *Halyomorpha halys*[J]. Journal of Pest Science, 2017, 90(4): 1219-1229. https://doi.org/10.1007/s10340-017-0862-z

**[7]** Kirkpatrick D M, Acebes-Doria A L, Rice K B, et al. Estimating monitoring trap plume reach and trapping area for nymphal and adult *Halyomorpha halys* (Hemiptera: Pentatomidae) in crop and non-crop habitats[J]. Environmental Entomology, 2019, 48(5): 1104-1112. https://doi.org/10.1093/ee/nvz093

**[8]** Morrison III W R, Cullum J P, Leskey T C. Evaluation of trap designs and deployment strategies for capturing *Halyomorpha halys* (Hemiptera: Pentatomidae)[J]. Journal of economic entomology, 2015, 108(4): 1683-1692. https://doi.org/10.1093/jee/tov159

**[9]** Morrison W R, Milonas P, Kapantaidaki D E, et al. Attraction of *Halyomorpha halys* (Hemiptera: Pentatomidae) haplotypes in North America and Europe to baited traps[J]. Scientific reports, 2017, 7(1): 1-11. https://doi.org/10.1038/s41598-017-17233-0

**[10]** Akotsen-Mensah C, Kaser J M, Leskey T C, et al. *Halyomorpha halys* (Hemiptera: Pentatomidae) responses to traps baited with pheromones in peach and apple orchards[J]. Journal of economic entomology, 2018, 111(5): 2153-2162. https://doi.org/10.1093/jee/toy200

**[11]** Nielsen A L, Dively G, Pote J M, et al. Identifying a potential trap crop for a novel insect pest, *Halyomorpha halys* (Hemiptera: Pentatomidae), in organic farms[J]. Environmental entomology, 2016, 45(2): 472-478. https://doi.org/10.1093/ee/nvw006

**[12]** Rice K B, Morrison III W R, Short B D, et al. Improved trap designs and retention mechanisms for *Halyomorpha halys* (Hemiptera: Pentatomidae)[J]. Journal of Economic Entomology, 2018, 111(5): 2136-2142. https://doi.org/10.1093/jee/toy185

**[13]** Morrison III W R, Park C G, Seo B Y, et al. Attraction of the invasive *Halyomorpha halys* in its native Asian range to traps baited with semiochemical stimuli[J]. Journal of Pest Science, 2017, 90(4): 1205-1217. https://doi.org/10.1007/s10340-016-0816-x

**[14]** Rice K B, Bedoukian R H, Hamilton G C, et al. Enhanced response of *Halyomorpha halys* (Hemiptera: Pentatomidae) to its aggregation pheromone with ethyl decatrienoate[J]. Journal of economic entomology, 2018, 111(1): 495-499. https://doi.org/10.1093/jee/tox316

**[15]** Short B D, Khrimian A, Leskey T C. Pheromone-based decision support tools for management of *Halyomorpha halys* in apple orchards: development of a trap-based treatment threshold[J]. Journal of Pest Science, 2017, 90(4): 1191-1204. https://doi.org/10.1007/s10340-016-0812-1

**[16]** Mathews C R, Blaauw B, Dively G, et al. Evaluating a polyculture trap crop for organic management of *Halyomorpha halys* and native stink bugs in peppers[J]. Journal of Pest Science, 2017, 90(4): 1245-1255. https://doi.org/10.1007/s10340-017-0838-z

**[17]** Blaauw B R, Morrison III W R, Mathews C, et al. Measuring host plant selection and retention of *Halyomorpha halys* by a trap crop[J]. Entomologia Experimentalis et Applicata, 2017, 163(2), 197-208. https://doi.org/10.1111/eea.12571

**[18]** Acebes-Doria A L, Morrison W R, Short B D, et al. Monitoring and biosurveillance tools for the brown marmorated stink bug, *Halyomorpha halys* (Stål)(Hemiptera: Pentatomidae)[J]. Insects, 2018, 9(3): 82. https://doi.org/10.3390/insects9030082

**[19]** Acebes-Doria A L, Agnello A M, Alston D G, et al. Season-long monitoring of the brown marmorated stink bug (Hemiptera: Pentatomidae) throughout the United States using commercially available traps and lures[J]. Journal of economic entomology, 2020, 113(1): 159-171. https://doi.org/10.1093/jee/toz240

**[20]** Tillman P G, Buntin G D, Cottrell T E. First Report of Seasonal Trap Capture for *Halyomorpha halys* (Hemiptera: Pentatomidae) and Native Stink Bugs in Central Georgia[J]. Journal of Entomological Science, 2017, 52(4): 455-459. https://doi.org/10.18474/JES17-56.1

**[21]** Rice K B, Cullum J P, Wiman N G, et al. *Halyomorpha halys* (Hemiptera: Pentatomidae) response to pyramid traps baited with attractive light and pheromonal stimuli[J]. Florida Entomologist, 2017, 100(2): 449-453. https://doi.org/10.1653/024.100.0207

**[22]** Chase K D, Stringer L D, Butler R C, et al. Multiple-Lure Surveillance Trapping for Ips Bark Beetles, Monochamus Longhorn Beetles, and *Halyomorpha halys* (Hemiptera: Pentatomidae)[J]. Journal of economic entomology, 2018, 111(5): 2255-2263. https://doi.org/10.1093/jee/toy190

**[23]** Bae S, Yoon Y, Jang Y, et al. Evaluation of an improved rocket traps, and baits combination for its attractiveness to hemipteran bugs in grass and soybean fields[J]. Journal of Asia-Pacific Entomology, 2017, 20(2): 497-504. https://doi.org/10.1016/j.aspen.2017.03.014

**[24]** Morrison III W R, Blaauw B R, Short B D, et al. Successful management of *Halyomorpha halys* (Hemiptera: Pentatomidae) in commercial apple orchards with an attract‐and‐kill strategy[J]. Pest management science, 2019, 75(1): 104-114. https://doi.org/10.1002/ps.5156

**[25]** Acebes‐Doria A L, Leskey T C, Bergh J C. Development and comparison of trunk traps to monitor movement of H alyomorpha halys nymphs on host trees[J]. Entomologia Experimentalis et Applicata, 2016, 158(1): 44-53. https://doi.org/10.1111/eea.12384

**[26]** Suckling D M, Levy M C, Roselli G, et al. Live traps for adult brown marmorated stink bugs[J]. Insects, 2019, 10(11): 376. https://doi.org/10.3390/insects10110376
